# A Study of Dysmenorrhea During Menstruation in Adolescent Girls

**DOI:** 10.4103/0970-0218.62586

**Published:** 2010-01

**Authors:** Anil K Agarwal, Anju Agarwal

**Affiliations:** Department of Community Medicine, G. R. Medical College, Gwalior-474 009, MP, India

**Keywords:** Adolescent girls, dysmenorrhea, physiological symptoms

## Abstract

**Research question:**

What is the prevalence of dysmenorrhea severity and its associated symptoms among adolescent girls?

**Objectives:**

(1) To study the prevalence of dysmenorrhea in high school adolescent girls of Gwalior. (2) To study the evidence of severity of the problem with associated symptoms and general health status.

**Study design:**

An explorative survey technique with a correlational approach.

**Setting and Participants:**

Nine hundred and seventy adolescent girls of age 15 to 20 years, studying in the higher secondary schools (Pre-University Colleges) of Gwalior.

**Statistical analysis:**

Percentages, Chi-square test, and Test-Retest Method.

**Results:**

The prevalence of dysmenorrhea in adolescent girls was found to be 79.67%. Most of them, 37.96%, suffered regularly from dysmenorrhea severity. The three most common symptoms present on both days, that is, day before and first day of menstruation were lethargy and tiredness (first), depression (second) and inability to concentrate in work (third), whereas the ranking of these symptoms on the day after the stoppage of menstruation showed depression as the first common symptoms. Negative correlation had found between dysmenorrhea and the General Health Status as measured by the Body surface area.

## Introduction

Adolescence is a transition period from childhood to adulthood and is characterized by a spurt in physical, endocrinal, emotional, and mental growth, with a change from complete dependence to relative independence. The period of adolescence for a girl is a period of physical and psychological preparation for safe motherhood. As the direct reproducers of future generations, the health of adolescent girls influences not only their own health, but also the health of the future population. Almost a quarter of India's population comprises of girls below 20 years.

One of the major physiological changes that take place in adolescent girls is the onset of menarche, which is often associated with problems of irregular menstruation, excessive bleeding, and dysmenorrhea. Of these, dysmenorrhea is one of the common problems experienced by many adolescent girls.

A dysmenorrhea incidence of 33.5% was reported by Nag (1982),(1) among adolescent girls in India. A study done in Sweden(2) showed that more than 50% of all menstruating women experience some discomfort. It has also been reported by a senior obstetrician that probably 5 – 10% of girls in their late teens suffer from severe spasmodic dysmenorrhea interrupting their educational and social life.(3)

The true incidence and prevalence of dysmenorrhea are not clearly established in India. In recent times, George and Bhaduri,(4) concluded that dysmenorrhea (87.87%) is a common problem in India. In Sweden the prevalence was >2–4%.(2) Similar findings had been reported by Jayashree and Jayalakshmi,(5) in rural married women of Andhra Pradesh. Dysmenorrhea has been estimated to be the greatest cause of time lost from work and school in the United States.(6)

A study of the prevalence of dysmenorrhea and its associated symptoms would provide evidence of the severity of the problem.

The study was carried out to estimate the prevalence of dysmenorrhea and its common symptoms, and determine the relationship between dysmenorrhea and the selected physiological parameters such as the body surface area and general health status, and to find the association between the dysmenorrhea status and the intensity of pain, with selected physiological symptoms.

## Materials and Methods

An explorative survey technique with a correlational approach was used for the study. The settings for the study were pre-university colleges (Higher Secondary School) in the Gwalior district. Only adolescent girls between 15 and 20 years, studying in the pre-university course were included in the study. A probability sampling method of the multistage cluster sampling technique was used to select the sample subjects. Three pre-university schools were selected and from this all the girls who met the sample criteria were included for data collection. The total sample size was 970. The data were collected from July to November 2002.

The tool developed was a semi-structured dysmenorrhea status questionnaire with a total of 14 items having a maximum score of ‘126’ and minimum score of ‘3’. The items included were presence and absence of dysmenorrhea, its frequency, intensity of pain, and symptoms experienced. A visual analog scale was used for measuring the pain intensity.

Content validity was established by a percentage of agreement of experts.

The test-retest method was employed to find the reliability, where ‘r’ was found to be 0.896. The body weight and height were measured by the standard scales after establishing the reliability of measurement.

## Results

### Prevalence of dysmenorrhea

Majority of the adolescent girls under study had experienced dysmenorrhea, that is, 698 out of 970 (71.96%), as shown in Table 1. Thus it can be said that dysmenorrhea is a very common problem among adolescent girls. Further analysis was conducted to find out how frequently they experienced dysmenorrhea. From Table 2 it can be seen that, the maximum number of girls, that is, 237 out of 698 girls (33.95%) experienced dysmenorrhea every month, and 118 (16.90%) experienced it in most of the months, and it was statistically highly significant (*P*<0.001).

**Table 1:** Frequencies and percentage of adolescent girls experiencing dysmenorrhea (Dysmenorrhea status)*

Adolescent girls with dysmenorrhea status	Frequency (N = 698)	Percentage
Dysmenorrhea every month	237	33.95
Most of the months	118	16.90
Occasionally	126	18.05
Rarely	217	31.09

*P* < 0.001;

* Total girls = 970, dysmenorrhea as ‘Yes’ groups (N) = 698, No dysmenorrhea groups = 272

**Table 2:** Percentage and rank of ten commonly occurring associated symptoms of menstruation and dysmenorrhea among adolescent girls

Symptoms	Day before menstruation		First day of menstruation		Day after stoppage of menstruation	
			
	%	Rank	%	Rank	%	Rank
Lethargy and tiredness	33.2	1	57.4	1	17.2	1
Irritability	30.9	2	32.9	7	7.3	10
Inability to concentrate on work	29.5	3	44.4	3	10.9	4
Feeling of heaviness in the lower abdomen	26.5	4	37.1	5	9.3	5
Nervousness	22.9	5	48.0	2	12.2	3
Depression	21.7	6	48.0	4	12.7	2
Anorexia	19.8	7	28.4	8	8.4	6
Loss of appetite	18.8	8	35.8	6	7.9	7
Sleeplessness	18.2	9	26.2	10	6.6	8
Headache	17.2	10	28.1	9	5.0	9

### Common symptoms experienced and their association with the dysmenorrhea status and the intensity of pain during menstruation

There were 23 symptoms grouped under four areas, such as, gastrointestinal symptoms (GI), psychological symptoms (PS), eliminational symptoms (ES), and other physical symptoms. The gastrointestinal symptoms were loss of appetite, increased appetite, nausea, vomiting, anorexia, and gaseous distension of abdomen. The psychological symptoms were depression, excitability, irritability, inability to concentrate on work, and nervousness. Elimination symptoms were: constipation, diarrhea, frequency of micturition, and profuse sweating. Other physical symptoms were lethargy and tiredness, headache, sleeplessness, increased sleep, fullness and tenderness of breasts, feeling of heaviness in the lower abdomen, pain and swelling in the ankle and knee joints, and swelling of face.

These 23 symptoms associated with menstruation anddysmenorrhea was ranked from the most commonly found at the day before menstruation and to the day after stoppage of menstruation. This ranking was done on the basis of the percentage of the girls who experienced each symptom, and the ten most commonly occurring symptoms on the day before, on the first day of menstruation, and the day after the stoppage of menstruation are presented in Table 2.

Three most common symptoms present on both days, that is, the day before and first day of menstruation were lethargy and tiredness (first), depression (second), and inability to concentrate on work (third), whereas, the ranking of these symptoms on the day of menstruation showed headache and anorexia as the eighth common symptom. Irritability was the second-most common symptom during the day before menstruation, and it become less on the first day of menstruation and the day after menstruation. Swelling of face was the least experienced problem by the girls, among the total of 23 listed symptoms.

The Chi-square tests were computed to find the association between the dysmenorrhea status, no dysmenorrhea status, and common symptoms experienced on the day before the onset of menstruation, on the first day of menstruation, and on the day after stoppage of menstruation. Similar statistical computation was also done between the intensity of pain and the listed symptoms. The average intensity of pain as measured by the visual analog scale, during menstruation, could be scored as 0, 1, 2, 3, 4, and 5, where 0 indicated no pain and 5 showed extreme pain. Tables 3–5, show the association between the symptoms and occurrence of dysmenorrhea and intensity of pain, with statistically significant difference, by the Chi square test.

**Table 3:** Association between psychological symptoms and intensity of pain during dysmenorrhea; dysmenorrhea groups (N) = 698

Variable	Conditions	Day before menstruation	First day of menstruation	Day after stoppage of menstruation
1	2	3	4	5
		Cases (%)	Cases (%)	Cases (%)
Depression		151 (21.6)+	308 (44.1)++	97 (13.9)+		
	P	3.94*	4.18**	2.9*
Excitability		19 (2.7)	25 (3.6)	8 (1.1)
	P	1.2	1.16	1.8
Irritability		216 (30.9)++	230 (32.9)++	51 (7.3)+
	P	3.6**	4.3**	2.3
Inability to concentrate on work		206 (29.5)++	310 (44.4)++	76 (16.9)+
	P	3.6**	4.3**	2.8*
Nervousness		160 (22.9)++	335 (48.0)++	89 (12.7)
	P	4.06**	4.28**	2.6

P= Intensity of pain found during dysmenorrhea on an average, measured as 0, 1,2,3,4, and 5; +/++= Chi square values indicating association between dysmenorrhea as ‘Yes’ and ‘No’ */**= Chi square values indicating intensity of pain

* *P* < 0.05 df (5)

+ *P* < 0.05 df (1)

** *P* < 0.01 df (5)

++ *P* < 0.01 df (1)

**Table 4:** Association between eliminational symptoms and intensity of pain during dysmenorrhea; dysmenorrhea groups (N) = 698

Variable	Conditions	Day before menstruation	First day of menstruation	Day after stoppage of menstruation
1	2	3	4	5
		Cases (%)	Cases (%)	Cases (%)
Constipation		98 (14.0)+	45 (6.4)	14 (2.0)
	P	2.8*	3.1*	1.9
Diarrhea		46 (6.6)	68 (9.7)+	32 (3.3)
	p	3.6**	4.1**	2.16
Frequency of micturition		1117 (16.8)++	155 (22.2)++	34 (4.9)+
	P	3.56	3.88**	1.2
Profuse sweating		117 (16.8)++	133 (19.0)++	26 (3.7)
	p	3.54**	4.02**	1.54

P= Intensity of pain found during dysmenorrhea on an average, measured as 0, 1, 2, 3, 4, and 5; +/++= Chi square values indicating association between dysmenorrhea as ‘Yes’ and ‘No’; */**= Chi square values indicating intensity of pain

* *P* < 0.05 df (5)

+ *P* < 0.05 df(1)

** *P* < 0.01 df (5)

++ *P* < 0.01 df(1)

**Table 5:** Association between other physical symptoms and intensity of pain during dysmenorrhea; dysmenorrhea group (N) = 698

Variable	Conditions	Day before menstruation	First day of menstruation	Day after stoppage of menstruation
1	2	3	4	5
		Cases (%)	Cases (%)	Cases (%)
Lethargy and tiredness		232 (33.2)++	401 (57.4)++	120 (17.1)+
	P	3.8**	4.28**	3.6**
Headache		120 (17.2)++	196 (28.1)++	35 (5.0)
	P	2.8*	3.9**	1.1
Sleeplessness		127 (18.2)++	183 (26.2)++	47 (6.6)
	P	2.8*	3.9**	0.8
Increased sleep		54 (7.7)+	105 (15.0)++	46 (6.6)+
	P	3.12*	4.16**	1.4
Fullness and tenderness of breast		104 (14.9)+	101 (14.5)+	34 (4.9)
	P	3.6*	3.4*	1.34
Feeling of heaviness in the lower abdomen		185 (26.5)++	259 (37.1)++	65 (9.3)+
	P	3.8**	4.2**	2.34*
Pain and swelling in the ankle and joint		40 (5.7)	47 (6.7)	19 (2.7)
	P	3.82*	3.96*	2.26*
Swelling of face		13 (1.9)	22 (3.1)	11 (1.6)
	P	0.9	3.2*	2.8*

P= Intensity of pain found during dysmenorrhea on an average, measured as 0, 1, 2, 3, 4, and 5; +/++= Chi square values indicating association between dysmenorrhea as /**= Chi square values indicating intensity of pain

* *P* < 0.05 df (5)

+ *P* < 0.05 df (1)

** *P* < 0.01 df (5)

++ *P* < 0.01 df (1)

### Gastrointestinal symptoms

On the day before menstruation, all but one symptom, that is, gaseous distension of abdomen was associated with the occurrence of dysmenorrhea, whereas, all the six symptoms were associated with intensity of pain during dysmenorrhea. On the first day of menstruation, except ‘increased appetite’ all other symptoms were associated with intensity of pain. On the day after the stoppage of menstruation, occurrence of dysmenorrhea and intensity of pain were associated with ‘anorexia’ and ‘vomiting’ only [Table 6].

**Table 6:** Association between gastrointestinal symptoms and intensity of pain during dysmenorrhea; dysmenorrhea groups (N) = 698

Variable	Conditions	Day before menstruation	First day of menstruation	Day after stoppage of menstruation
1	2	3	4	5
		Cases (%)	Cases (%)	Cases (%)
Loss of appetite		131 (18.8)+	250 (35.8)++	55 (7.9)
	P	2.8*	4.03**	0.8
Increased appetite		77 (11.0)+	55 (7.9)	28 (4.0)
	P	1.34*	2.3	1.2
Nausea		118 (16.9)++	147 (21.1)++	26 (3.7)
	P	3.9**	4.18**	1.9
Vomiting		75 (10.7)+	90 (12.9)+	23 (3.3)
	P	4.01**	3.84**	2.6*
Anorexia		138 (19.8)++	198 (28.4)++	59 (8.4)
	P	4.24**	4.48**	3.36*
Gaseous distension of abdomen		34 (4.9)	45 (6.4)	12 (1.7)
	P	1.8	1.9	0.9

P=Intensity of pain found during dysmenorrhea on an average, measured as 0, 1, 2, 3, 4, and 5; +/++ Chi square values indicating association between dysmenorrhea as ‘Yes” and “No’; */**Chi square values indicating intensity of pain

* *P* < 0.05 df (5)

+ *P* < 0.05 df (1)

** *P* < 0.01 df (5)

++ *P* < 0.01 df (1)

### Psychological symptoms

All the psychological symptoms except excitability were closely associated with the occurrence of dysmenorrhea and intensity of pain, both on the day before and after the stoppage of menstruation. On the day after the stoppage of menstruation the symptom of ‘irritability’ was found to be associated with the occurrence of dysmenorrhea, and ‘depression’ and ‘inability to concentrate on work’ was found to be associated with the intensity of pain [Table 3].

### Eliminational symptoms

Significantly more girls with dysmenorrhea experienced constipation, frequency of micturition, and profuse sweating on the day before menstruation, while they experienced diarrhea, frequency of micturition, and profuse sweating on the first day of menstruation. None of the eliminational symptoms were associated with the occurrence of dysmenorrhea on the day after stoppage of menstruation, whereas, all the eliminational symptoms were found to be highly associated with the intensity of pain, both on the day before and on the first day of menstruation. These findings suggest that the eliminational symptoms, which appear just before or during menstruation, disappear with the stoppage of menstruation [Table 4].

### Other physical symptoms

Lethargy and tiredness were persistent problems in girls with dysmenorrhea. They were also associated with intensity of pain on all three days of menstruation. All the other physical symptoms except increased sleep were associated with dysmenorrhea occurrence and intensity of pain on both days, that is, day before and on the first day of menstruation, whereas, intensity of pain was associated with the symptoms of ‘pain and swelling in the ankle and knee joints’ and swelling of face on the day after the stoppage of menstruation also. ‘Fullness and tenderness of breast’, ‘feeling of heaviness in the lower abdomen’ and ‘headache’ on the day after the stoppage of menstruation was associated with dysmenorrhea [Table 5].

### Relationship between dysmenorrhea and body surface area

A simple correlation ‘r’ was computed to find out the relationship between dysmenorrhea in adolescent girls and their body surface area. Body surface area (BSA) was calculated by using the following formula given by Dubois, and Dubois.(16)

BSA=Ht.(cms)×Wt.(kgs)100×60

The correlation value obtained was negative (0.023), which was not significant at 0.05 levels. However, the negative value showed that there was a low, negative correlation between dysmenorrhea and the general health status as measured by the Body Surface Area. Therefore, it can be interpreted that the severity of dysmenorrhea and the physiological variables, that is, Body Surface Area are not related.

## Discussion

The findings of the present study showed a high prevalence of dysmenorrhea, that is, 71.96% among adolescent girls of Gwalior. Similar findings were reported by George and Bhaduri (87.87%),(4) Mckay and Diem (67%),(7) Jayashree and Jayalakshmi (74%),(5) Andersch and Milson (80%),(2) Sundel *et al*, (67%),(8) and Harlow and Park (71.6%).(9) Comparatively lower incidences had been reported by Nag (33.84%).(1)

The Gastrointestinal, Psychological, Eliminational, and other Physical Symptoms, similar to the ones found associated with dysmenorrhea in the present study, were reported by George and Bhaduri,(4) Agarwal and Agarwal,(10) Jarret *et al*,(11) Lewis,(12) Wenkz,(13) and Parsens and Sammer.(14)

The findings of the recent study, of there not being a relationship between the severities of dysmenorrhea and body surface area contradict the explanations by Jeffcoate(15) and Dawn (1990),(3) which states that general ill health is one of the etiological causes of dysmenorrhea. However, it supports the findings of George and Bhaduri,(4) and Sundell *et al*.(8)

## Conclusion

From the study it can be concluded that dysmenorrhea is a very common problem among adolescent girls, and they experience a number of physical and emotional symptoms associated with dysmenorrhea, and with the increased intensity of pain in occurrence of dysmenorrhea the probability of experiencing these symptoms is also increased.

Adolescent girls, almost always, silently suffer the pain by dysmenorrhea and the discomfort associated with it due to lack of knowledge about reproductive health. It is probable that this also affects their academic performance. The findings of this study thus indicate the enormity of the problem and the need for appropriate intervention through a change in lifestyle.
